# The correlation between HER-2 expression and the CEUS and ARFI characteristics of breast cancer

**DOI:** 10.1371/journal.pone.0178692

**Published:** 2017-06-01

**Authors:** Xiao-Yan Wang, Qiao Hu, Meng-Yuan Fang, Yan He, Hai-Ming Wei, Xue-Xue Chen, Bing Zhang

**Affiliations:** 1 Department of Ultrasound, the People's Hospital of Guangxi Zhuang Autonomous Region, Nanning, Guangxi, China; 2 Department of Pathology, the People's Hospital of Guangxi Zhuang Autonomous Region, Nanning, Guangxi, China; University of South Alabama Mitchell Cancer Institute, UNITED STATES

## Abstract

**Objective:**

The purpose of the present study was to explore the correlation between the contrast-enhanced ultrasound (CEUS) and acoustic radiation force impulse (ARFI) characteristics of breast cancers and the expression of human epidermal growth factor receptor 2 (HER-2).

**Methods:**

HER-2 expression levels in the tumor masses of 167 clearly diagnosed cases of breast cancer were measured and analyzed. The enhancement features and time intensity curve (TIC) of CEUS and virtual touch tissue imaging (VTI) and virtual touch tissue quantification (VTQ) technology of ARFI were employed to analyze the relationship between HER-2 expression and the CEUS and ARFI characteristics of breast cancer.

**Results:**

(1) Statistically significant differences in the distribution of the contrast agent, perforator blood flow, the overranging phenomenon and perfusion defects between the study groups with different HER-2 expression levels (*P* < 0.05) were observed on CEUS. In addition, statistically significant differences in the TIC peak time (PT), slope of the ascending branch (K) and area under the curve (AUC) were found in the groups expressing different levels of HER-2 (*P* < 0.05). In contrast, the degree of contrast agent enhancement and TIC peak intensity (PI) were found to be independent of the expression status of HER-2, as they were not statistically significant (*P* > 0.05). (2) Statistically significant differences in the VTQ results between the groups with different HER-2 expression levels were found (*P* < 0.05). However, no statistically significant differences in VTI image characteristics were detected between the groups expressing different levels of HER-2 (*P* > 0.05).

**Conclusion:**

A correlation was found between the CEUS and ARFI characteristics of breast cancer and HER-2 expression levels. This correlation was principally reflected in perfusion defects, perforator blood flow, PI, PT, K and VTQ.

## Introduction

Breast cancer is a common malignancy in females. Since 1995, the incidence of breast cancer has continued to increase at a rate of 1.5% per year [1]. Thus, the early detection, diagnosis and treatment of breast cancer are high priority issues. New diagnostic technologies including contrast-enhanced ultrasound (CEUS) and acoustic radiation force impulse (ARFI) imaging have gradually matured and are now widely applied in breast cancer research. CEUS provides higher-quality images of the micro-vessels in tumor masses than conventional two-dimensional (2D) color Doppler ultrasound. In addition, CEUS allows for real-time continuous monitoring of the entire blood perfusion process and improves the accuracy of classifying malignant versus benign masses [2–4]. Elastography helps to determine the nature of a mass via differences in the elastic modulus between the mass and its surrounding normal tissues. Studies by Nightingale et al. have confirmed that the strain graphs generated by ARFI technology correspond well to tissue structures on 2D ultrasound [5,6]. In addition, the contrast of ARFI images is superior to that of 2D ultrasound images. The present study aimed to explore the correlation between various CEUS/ARFI indices and the expression status of human epidermal growth factor receptor 2 (HER-2), thereby providing a scientific basis for the clinical diagnosis, treatment and prognosis of breast cancer.

## Materials and methods

### Research subjects

The study was approved by the Ethical Committee of the People’s Hospital of Guangxi Zhuang Autonomous Region and written informed consent was obtained from all patients. A total of 167 24–82-year-old female breast cancer patients were consecutively included in the study. The patients had not received chemotherapy or any other treatments between September 2014 and May 2016. The patients underwent CEUS and ARFI examinations at our hospital. In addition, the patients were all pathologically confirmed to have breast cancer and subjected to post-surgery immunohistochemical examination. Due to the limitations of the ARFI sampling frame, masses less than 6 mm in diameter were excluded from the study. The mean age of the patients was 58.2 ± 10.3 years.

### Instruments and examination methods

**Fig 1:**
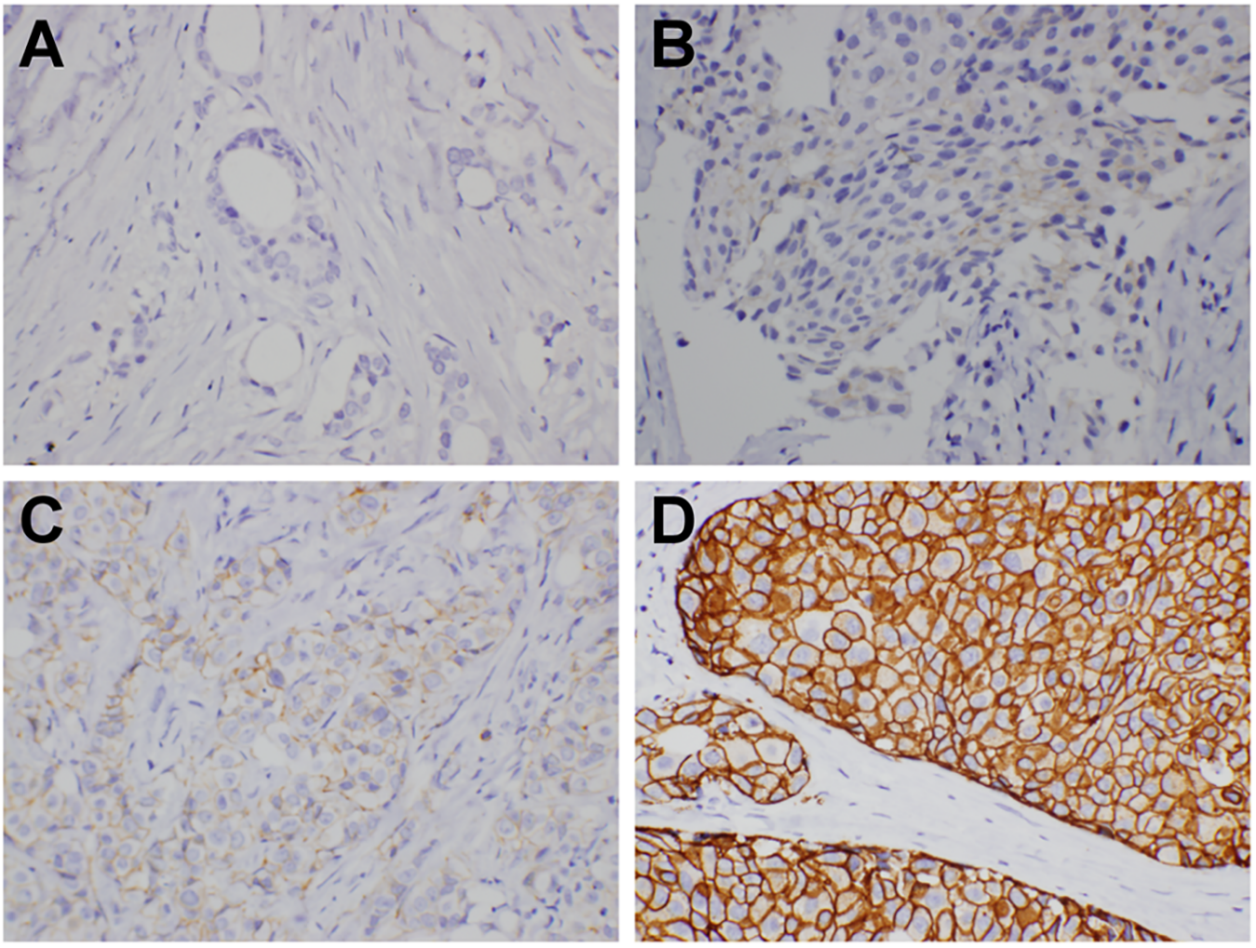
CEUS characteristics of HER-2 (+++) breast cancer. 1a, perforator vessels (indicated by arrow);1b,overranging;1c, perfusion defects (indicated by arrow); 1d,TIC produced by CEUS (K, PT, AUC, etc.).

**Fig 2:**
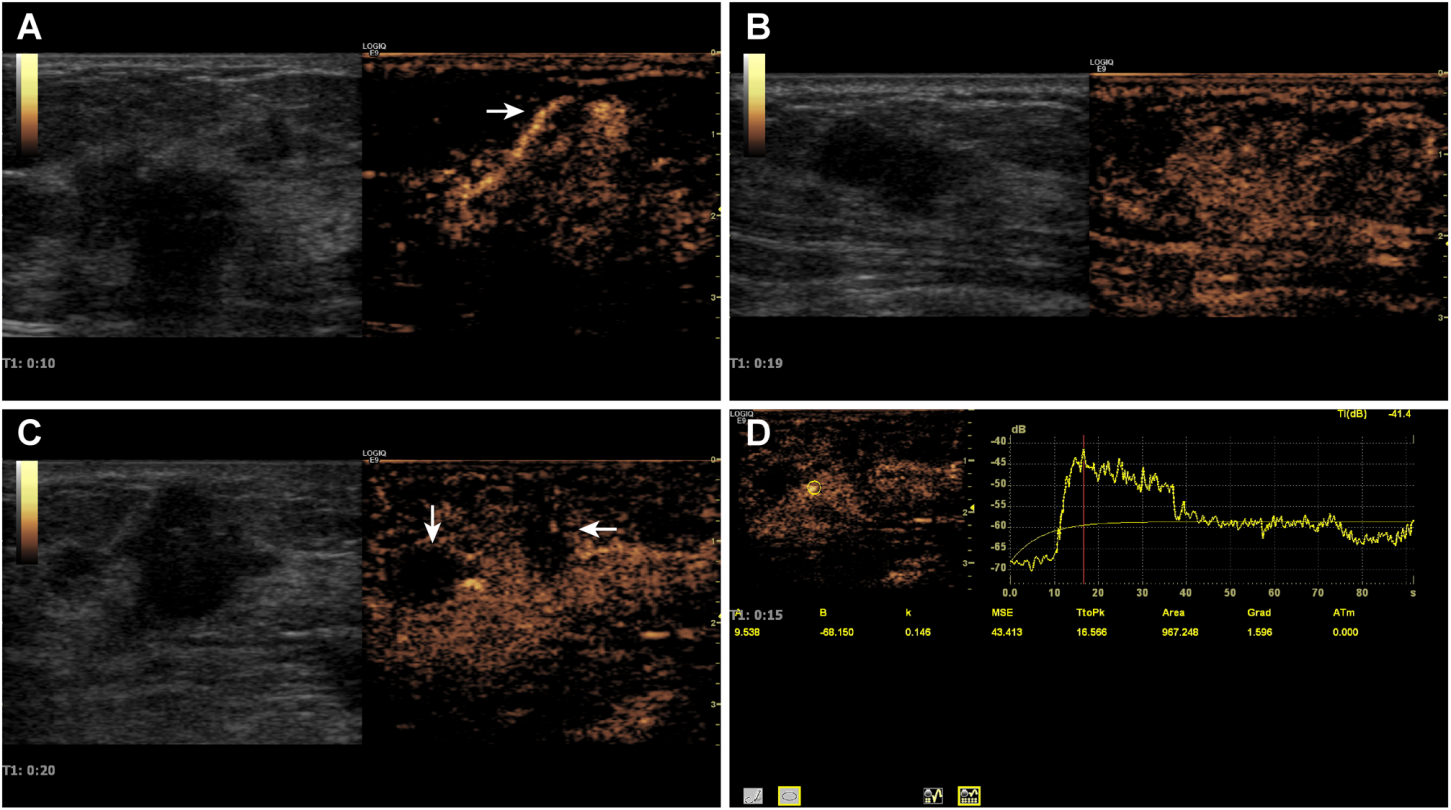
ARFI characteristics of HER-2 (+++) breast cancer. 2a, 2D color Doppler ultrasound image; 2b, VTI image; 2c, VTQ image.

**Fig 3:**
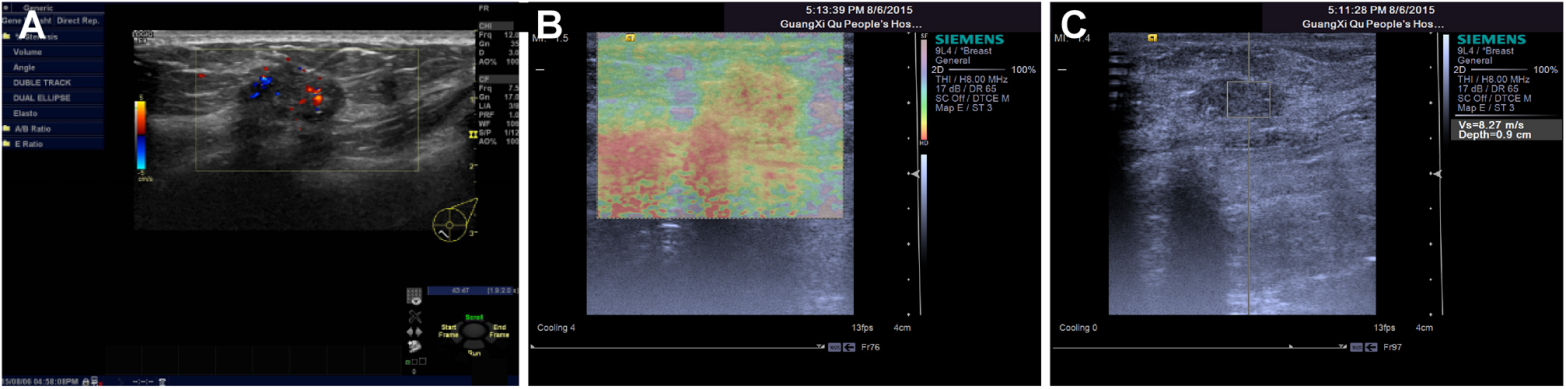
Immunohistochemical staining results (× 400). 3a, HER-2 (-); 3b, HER-2 (+); 3c, HER-2 (++); 3d, HER-2 (+++).

1) CEUS was performed using the GE LOGIQ E9 Diagnostic Ultrasound System. The high-frequency probe had a frequency of 15.0 MHz, the contrast probe had a frequency of 9.0 MHz, and the mechanical index was 0.08. The lesion section with the richest blood flow was selected before switching to the contrast mode. The patients were then asked to engage in quiet breathing. The contrast agent SonoVue (2–4 ml) was administered by bolus injection via the cubital vein, and continuous real-time observation was performed on the dynamic process of lesion perfusion. The minimal dynamic image acquisition time was 3 min. After the completion of CEUS, the features of the CEUS images, including the distribution of the contrast agent, the degree of enhancement, the perforator vessels and the perfusion defects, were analyzed (Fig 1A, 1B and 1C). In addition, a time-intensity curve (TIC) analysis was prepared. The regions of interest were set to include the mass regions with a high degree of contrast agent-induced signal enhancement, and the corresponding TICs were plotted. The peak time (PT), peak intensity (PI), slope of the ascending branch (K) and area under the curve (AUC) were recorded (Fig 1D).

2) The Siemens ACUSON S2000 diagnostic ultrasound system was used to implement ARFI imaging technology. The ultrasound system was equipped with ARFI imaging software and the 9 L4 high-frequency probe (7.0–12.0 MHz). The section that best represented the characteristics of a lesion and its surrounding tissues was selected for analysis. The image was stabilized before switching to the ARFI mode. Virtual touch tissue imaging (VTI) of the ARFI was performed (Fig 2B). The sampling frame was then adjusted to cover the lesion and its surrounding tissues. The lesions were largely examined in triplicate, and the best images were selected and stored for subsequent analysis. A 5-pointscoring system was used to determine whether a breast lesion was benign or malignant [7,8]. A breast lesion that scored less than 4 points was diagnosed as a benign lesion, whereas one that scored 4 or higher was diagnosed as a malignant lesion. The virtual touch quantification (VTQ) technique was subsequently applied (Fig 2C). The sampling frame was placed in the region of interest, and the elastic shear-wave velocity (SWV) of the selected region of interest was measured in meters per second (m/s). The SWV was measured 5–7 times consecutively, and the average value of the SWV was calculated. The VTQ results were stored for subsequent analysis. In some cases, the SWV values were displayed as ‘X.XX m/s’ rather than as specific numerical values, even after repeated measurement. Excluding potential method and operational errors, the true SWV values were assumed to exceed the upper measurement range limit (9 m/s), and the SWV values were recorded as ‘9 m/s’ [9].

3) The EnVision™ immunohistochemical method was used by pathologists in our hospital to analyze the HER-2 expression levels. They had no knowledge of the conclusions of the imaging examinations and classified HER-2 expression into four distinct levels (-, +, ++ and +++), as shown in Fig 3). In addition, HER-2 (-) and HER-2 (+) are classified as HER-2 negative, while HER-2 (+++) is classified as HER-2 positive. HER-2 (++) is considered to be a borderline result. In such cases, fluorescence in situ hybridization (FISH) was performed to examine the amplification of HER-2. If no HER-2 amplification was found, HER-2 (++) was classified as HER-2 negative. If HER-2 amplification occurred, HER-2 (++) was classified as HER-2 positive [10].

### Statistical analysis

The data were analyzed using the SPSS16.0 statistical package. The measured data that met the homogeneity of variance assumption were subjected to analysis of variance (ANOVA or Two-tailed t-test), while the count data were analyzed using a chi-square test (Fisher's exact test was employed if the theoretical frequency (T) was less than 1, or if the number of cells with a T value between 1 and 5 accounted for more than 1/5 of the total number of cells). P values of less than 0.05 were considered to be statistically significant.

## Results

1. Histopathological classification

1) Type of breast cancer: there were 121 cases of infiltrating ductal carcinoma, 42 cases of intraductal carcinoma, 2 case of neuroendocrine cancer and 2 cases of papillary carcinoma.

2) Immunohistochemical phenotype: there were 71 cases of Her-2 (-) breast cancer (42.5%), 37 cases of Her-2 (+) breast cancer (22.1%), 26 cases of Her-2 (++) breast cancer (15.6%) and 33 cases of Her-2 (+++) breast cancer (19.8%). FISH test confirmed there were 4 cases classified as HER-2 negative, and 22 cases classified as HER-2 positive within Her-2 (++) group. Finally, 112 cases were classified as HER-2 negative breast cancer and 55 cases were classified as HER-2 positive breast cancer.

2. The following comparison of the CEUS characteristics of the various breast cancer groups expressing different levels of HER-2 was conducted

1) Contrast enhancement characteristics: the distribution of the contrast agent, perforator blood flow, overranging and perfusion defects on CEUS were found to be correlated with HER-2 levels. As shown in Table 1 and Table 2, statistically significant differences were detected between the HER-2 negative groups and the HER-2 positive groups in the distribution of the contrast agent, perforator blood flow, perfusion defects, and the overranging phenomenon (*P* < 0.05). In addition, statistically significant differences were detected between the HER-2 (-) group and all the other groups in the distribution of the contrast agent, perfusion defects and the overranging phenomenon (*P* < 0.05). Statistically significant differences in perforator blood flow were noted between the HER-2 (+++) and HER-2 (-) groups and also between the HER-2 (+++) and HER-2 (+) groups (*P* < 0.05). In contrast, no statistically significant differences were found between the remaining groups in terms of the distribution of the contrast agent, perforator blood flow, overranging and perfusion defects (*P* > 0.05). The degree of contrast agent enhancement was found to be independent of the expression status of HER-2, as these differences were not statistically significant (*P* > 0.05).

**Table 1:** Relationships between contrast enhancement characteristics on CEUS and HER-2 expression.

HER-2	Distribution of contrast agent		Degree of enhancement		Perforator vessels		Overranging		Perfusion defects	
	Uniform	Non-uniform	Hyperenhanced	Isoenhanced	Present	Absent	Present	Absent	Present	Absent
negative	43	67	93	17	42	68	57	53	39	71
positive	8	49	48	9	38	19	48	9	45	12
*P* value	0.001		0.955		0.000		0.000		0.000	

**Table 2:** Contrast enhancement characteristics on CEUS with different HER-2 levels.

HER-2	Distribution of contrast agent		Degree of enhancement		Perforator vessels		Overranging		Perfusion defects	
	Uniform	Non-uniform	Hyperenhanced	Isoenhanced	Present	Absent	Present	Absent	Present	Absent
-	32	39#&△	59	12	29	42	37	34#&△	28	43#&△
+	7	30	31	6	15	22	27	10	23	14
++	6	20	19	7	14	12	20	6	18	8
+++	4	29	27	6	22	11*#	27	6	24	9

Note

* indicates *p* < 0.05 compared with the—group

# indicates *p* < 0.05 compared with the + group

& indicates *p* < 0.05 compared with the ++ group

△ indicates *p* < 0.05 compared with the +++ group.

2) Quantitative TIC analysis: Statistically significant differences were found in the PT, AUC, and K values between the HER-2 negative and the HER-2 positive groups. The differences between the PT and K values of the HER-2 (+++) and HER-2 (-) groups were statistically significant (*P* < 0.05). In addition, statistically significant AUC differences were detected between the HER-2 (+++) and HER-2 (-) groups as well as between the HER-2 (+++) and HER-2 (+) groups (*P* < 0.05). As the HER-2 levels increased, the PI values increased accordingly. However, no statistically significant difference in PI was found between the groups (*P* > 0.05). The TIC results are summarized in Table 3 and Table 4.

**Table 3:** Relationships between quantitative CEUS*-*TIC indices and HER-2 expression.

HER-2	PT(s)	PI (dB)	K	AUC
negative	23.648±4.994	-47.734±5.578	0.104±0.022	823.454±280.572
positive	20.354±3.560	-46.340±4.200	0.118±0.022	1034.355±281.272
*P* value	0.000	0.185	0.003	0.000

Variables are expressed as Mean ± SD

**Table 4:** Quantitative CEUS*-*TIC indices with different HER-2 levels.

HER-2	PT(s)	PI (dB)	K	AUC
**-**	23.228 ± 4.594	-49.633 ±6.591	0.104 ± 0.020	800.704 ± 270.535
**+**	23.046 ± 4.239	-47.374 ± 6.982	0.107 ± 0.024	878.239 ± 276.734
**++**	21.476 ± 4.654	-47.710 ± 5.221	0.107 ± 0.025	994.662 ± 272.095
**+++**	20.143 ± 3.457*	-46.134 ± 5.907	0.122 ± 0.023*	1089.988 ± 287.094*#

Variables are expressed as Mean ± SD

* indicates *p* < 0.05 compared with the—group

# indicates *p* < 0.05 compared with the + group.

3. The following comparison of the ARFI characteristics of the various breast cancer groups expressing different levels of HER-2 was conducted

As shown in Table 5 and Table 6, there were no statistically significant differences between the groups in the features of the VTI images (*P* > 0.05). In contrast, statistically significant differences were detected between the VTQ results of the HER-2 negative and the HER-2 positive groups, HER-2 (+++) and HER-2 (-) groups, and between the HER-2 (+++) and HER (+) groups (*P* < 0.05).

**Table 5:** Relationship between ARFI indices and HER-2 expression.

HER-2	VTI		VTQ (m/s)
	< 4	≥ 4	
negative	17	93	5.747±1.954
positive	8	49	6.912±1.575
*P* value	0.807		0.002

VTQ variables are expressed as Mean ± SD

**Table 6:** Quantitative ARFI indices with different HER-2 levels.

HER-2	VTI		VTQ (m/s)
	< 4	≥ 4	
-	17	54	5.553 ± 2.072
+	9	28	5.907 ± 2.012
++	8	18	6.366 ± 1.721
+++	10	23	7.183 ± 1.553*#

VTQ variables are expressed as Mean ± SD

* indicates *p* < 0.05 compared with the—group

# indicates *p* < 0.05 compared with the + group.

## Discussion

Recent advances in medical science have improved breast cancer diagnosis and treatment. Currently, we are able to not only comprehend the pathological types of breast cancer but also examine various immunohistochemical indicators of breast cancer such as the estrogen receptor (ER), the progesterone receptor (PR), HER-2 and Ki67. HER-2 gene is now a hotspot in breast cancer research. HER-2 expression has been positively correlated with vascular endothelial growth factor (VEGF) [11]. Therefore, high HER-2 expression promotes tumor neovasculature formation and increases the invasiveness of tumor cells. In patients, HER-2 gene overexpression indicates a high degree of tumor malignancy, low survival rate, high recurrence rate and high potential for lymph node metastasis. Therefore, the determination of HER-2 expression levels is of great significance for the selection of treatment regimens. In normal tissues, the HER-2 gene is either unexpressed or expressed at trace levels; however, the HER-2 gene is overexpressed in breast cancer. Hence, breast cancer is sensitive to targeted drug therapies [12]. A study conducted by Cobleigh et al. showed that higher levels of HER-2 overexpression were associated with enhanced effectiveness of the commonly-used targeted drug Herceptin [13].

CEUS is a novel diagnostic technique. In contrast with 2D ultrasound, CEUS can reveal tumor microvessels with low volume and blood flow velocity. CEUS greatly improves diagnostic accuracy and contributes significantly to the development of ultrasound diagnostics [14]. The present study showed that HER-2 overexpression was related to the distribution of the contrast agent, perforator blood flow, overranging and perfusion defects. In addition, the volume of the contrast agent and overrange distributions, as well as the number of perfusion defects and perforator vessels, were significantly lower in the HER-2(-) group when compared with those in all other groups. These results are consistent with the research findings of Li et al. [15]. The HER-2 gene can stimulate the formation of tumor neovasculature. As the number of blood vessels in a tumor increases, the tumor becomes nourished, and the tumor volume increases rapidly with the tumor cells’ increased oxygen consumption. As a result, a portion of the tumor tissues undergo liquefactive necrosis due to an insufficient supply of nutrients [16]. Therefore, perfusion defects are often detected in HER-2 positive tumors during CEUS. Whether or not the contrast agent is uniformly distributed may be related to the tissue structure and uniformity of the blood vessel distribution inside the tumor mass [17]. Perfusion defects also increase the probability of an asymmetrical distribution of the contrast agent. The formation of perforator vessels may involve the growth of tumor neovasculature, which generally extends from the tumor periphery toward the tumor, as well as the infiltration of internal tumor vessels into the tumor periphery. Breast cancers overexpressing the HER-2 gene are highly invasive, exhibiting an infiltrative growth pattern. In addition, in these types of breast cancer, the growth of tumor blood vessels occurs more rapidly than changes in tumor size and morphology. Therefore, the range of contrast agent-induced enhancement in CEUS is greater than the mass size shown in 2D ultrasound. In the present study, the CEUS TIC analysis showed that statistically significant differences in PT, K and AUC were associated with different levels of HER-2 expression. When the HER-2 gene was overexpressed, the number of tumor blood vessels increased accordingly. Therefore, compared with the HER-2 negative group, the HER-2 overexpressing group required less time for peak enhancement and was associated with an increase in K value after the contrast agent entered the tumor. In contrast, the HER-2 overexpressing group was associated with a prolonged wash-out time and a relatively flattened descending branch, resulting in a higher AUC than the HER-2 negative group. No statistically significant differences were found between the TIC PI values of the HER-2 negative and HER-2 overexpressing groups, possibly due to the patients’ diverse selected regions of interest and levels of systemic circulation. There were no differences in the degree of contrast agent enhancement between the HER-2 negative and HER-2 overexpressing groups. In addition to the reasons described above, the lack of difference in the degree of contrast enhancement might also be due to the inherent subjectivity of the judgment process.

ARFI is an ultrasound elastography technique. When ultrasonic beams act on tissues, longitudinal compression and transverse vibration occur, resulting in the generation of shear waves. In contrast with other elastography techniques, ARFI tissue reactions do not depend on applying an external compressive force but utilize the texture of the tissue itself. VTI is an elastography technique based on longitudinal displacement. VTI generates grayscale or color images to reflect the degree of tissue elasticity. Compared with 2D ultrasound, VTI not only evaluates the nature of the masses in a more accurate and intuitive manner [18] but also indirectly reflects the degree and scope of the involvement of the surrounding tissues [19]. During VTQ, tissues are exposed to a pulse wave that generates lateral shear waves. The shear wave velocity (SWV) is obtained using the peak time difference between the adjacent shear waves and their wavelengths. Higher SWV values are associated with stiffer tissues [20]. In the present study, we compared the VTQ SWVs of the HER-2 (+++) and HER-2 (-) groups as well as those of the HER-2 (+++) and HER-2 (+) groups. These VTQ SWV differences were found to be statistically significant. The HER-2 gene promotes the proliferation and differentiation of tumor cells as well as the active hyperplasia of stromal cells, resulting in increased tumor mass stiffness. In contrast, no statistically significant differences were found in the VTI results of the different groups studied. The formation of a breast mass is complex in nature. The stiffness of a breast mass is affected by a number of factors in addition to the HER-2 gene. Moreover, the VTI technique relies substantially on the subjective judgments of physicians. Therefore, subtle changes in the HER-2 levels between the different study groups may not have given rise to significantly different VTI results. On the present study, we found no significant differences between the HER-2 (++) group and the other groups in PT, K value, VTQ value and other parameters. To a certain extent, this result demonstrates the peculiarity of the HER-2 (++) group, which might include both HER-2 negative and positive breast cancers.

There are two limitations to our study. First, it lacks evaluation of the intra- and inter-observer variability in the present study. Additional investigation is needed regarding this issue. Furthermore, the potential confounding factors (e.g. tumour grade, ER, PR status) in the analysis of CEUS/ARFI indices required further studies in breast cancer patients.

## Conclusion

The present study highlights certain differences in the CEUS and ARFI characteristics of breast cancer patients with different expression levels of HER-2. These differences are mainly reflected in perfusion defects and perforator blood flow, as well as in PI, PT, K and VTQ values. However, the promotion of breast tumor angiogenesis, proliferation and differentiation is not solely dependent on HER-2 expression. In addition, we found that imaging examinations are affected by several factors. At present, HER-2 can only be employed for preliminary clinical judgment. Further studies are required to confirm the feasibility of assessing the effects of subtle changes in HER-2 levels by imaging examination and applying them to guide clinical treatment.
